# The Challenges and Perspectives of the Integration Between Virtual and Augmented Reality and Manual Therapies

**DOI:** 10.3389/fneur.2021.700211

**Published:** 2021-06-30

**Authors:** Francesco Cerritelli, Marco Chiera, Marco Abbro, Valentino Megale, Jorge Esteves, Alberto Gallace, Andrea Manzotti

**Affiliations:** ^1^Foundation COME Collaboration, Pescara, Italy; ^2^Softcare Studios, Rome, Italy; ^3^Department of Psychology, Bicocca University, Milan, Italy; ^4^RAISE Lab, Foundation COME Collaboration, Milan, Italy; ^5^SOMA Istituto Osteopatia Milano, Milan, Italy

**Keywords:** balance, cybersickness, head-mounted display, multisensory integration, presence, simulation, touch

## Abstract

Virtual reality (VR) and augmented reality (AR) have been combined with physical rehabilitation and psychological treatments to improve patients' emotional reactions, body image, and physical function. Nonetheless, no detailed investigation assessed the relationship between VR or AR manual therapies (MTs), which are touch-based approaches that involve the manipulation of tissues for relieving pain and improving balance, postural stability and well-being in several pathological conditions. The present review attempts to explore whether and how VR and AR might be integrated with MTs to improve patient care, with particular attention to balance and to fields like chronic pain that need an approach that engages both mind and body. MTs rely essentially on touch to induce tactile, proprioceptive, and interoceptive stimulations, whereas VR and AR rely mainly on visual, auditory, and proprioceptive stimulations. MTs might increase patients' overall immersion in the virtual experience by inducing parasympathetic tone and relaxing the mind, thus enhancing VR and AR effects. VR and AR could help manual therapists overcome patients' negative beliefs about pain, address pain-related emotional issues, and educate them about functional posture and movements. VR and AR could also engage and change the sensorimotor neural maps that the brain uses to cope with environmental stressors. Hence, combining MTs with VR and AR could define a whole mind-body intervention that uses psychological, interoceptive, and exteroceptive stimulations for rebalancing sensorimotor integration, distorted perceptions, including visual, and body images. Regarding the technology needed to integrate VR and AR with MTs, head-mounted displays could be the most suitable devices due to being low-cost, also allowing patients to follow VR therapy at home. There is enough evidence to argue that integrating MTs with VR and AR could help manual therapists offer patients better and comprehensive treatments. However, therapists need valid tools to identify which patients would benefit from VR and AR to avoid potential adverse effects, and both therapists and patients have to be involved in the development of VR and AR applications to define truly patient-centered therapies. Furthermore, future studies should assess whether the integration between MTs and VR or AR is practically feasible, safe, and clinically useful.

## Introduction

Virtual reality (VR) and augmented reality (AR) have recently drawn professionals and patients' attention in several fields, including psychology, physical, and neurological rehabilitation, and surgery. In particular, the development of low-cost head-mounted displays (HMDs), which can be associated with smartphones, gaming consoles, personal computers, or workstations, brought VR and AR outside research laboratories toward a vast audience of users (1, 2). VR and AR typically use input devices to gather information about the user (e.g., the position of the body and its kinetic information) and the surrounding environment, and output devices to send the user sensory information (e.g., images, vibrations, and sounds) (1).

VR aims to create an environment that the users feel as realistic and coherent: they should experience “presence”—the illusion of “being there” in the simulated environment and the feeling that what is happening is plausible. Indeed, the simulated events have to follow precise physical laws, satisfy psychosocial expectations, and synchronize with the user's actions (2). AR aims to superimpose virtual information on the physical world: just like VR, AR should synchronize the virtual simulation with the real world to give a fair degree of realism and a sense of presence (1). Since the virtual objects extend the real environment (the users “are already there”), the sense of presence in AR could be better defined as “informed continuity.”

The efficacy of both VR and AR lies, thus, in the creation of a coherent simulation: the users must experience the same physical laws during the whole simulation and perceive synchronicity between their (re)actions and the virtual stimuli. Once accustomed to the simulation, the users must see congruence between what is happening and their expectations (1). If the simulated environment lacked coherence, users would feel it as non-realistic: they would live a poor experience, the simulation would fail to induce positive effects, and adverse effects such as cybersickness (i.e., the feeling of discomfort and malaise due to the mismatch between observed and expected sensory signals) could occur. Note that some forms of sickness, e.g., motion sickness, can occur even if users experience presence: in fact, for both presence and cybersickness to occur, the patients have to feel the simulation as realistic (1, 3).

In the last decade, VR and AR applications in the medical and psychological fields have increased: they are used for educational purposes, surgical training and procedures, neuromotor rehabilitation, and anxiety treatments (1, 4). However, more high-quality research is required to include these technologies in healthcare curricula efficiently (5).

Manual therapies (MTs) are touch-based approaches, such as various types of massage or osteopathic manipulative treatment (OMT), that involve the manipulation of tissues (6). Although more rigorous research is required, several papers showed MTs might influence the body's myofascial system, affect local and systemic circulation, improve sleep, and reduce pain (6–9). Despite VR and AR involvement in physical therapy, the literature lacks papers assessing the relationship between MTs and VR or AR, to the best of our knowledge. The published trials about VR and AR in the context of manual approaches discuss VR and AR integration with physical rehabilitation (e.g., physiotherapy for stroke patients) (10, 11), which goes beyond touch or manipulation *per se*. Hence, the interaction of MTs with VR and AR remains somehow unknown.

Therefore, after briefly reviewing the effects of MTs, VR, and AR in healthcare, the present paper aims to propose how these approaches, in particular, MTs and VR, could be combined for improving patient care. Indeed, integrating the tactile, proprioceptive, and interoceptive sensations elicited by MTs with the (mainly visual-auditory) simulated VR experience could harness the benefits and overcome the limits of both. Their integration's positive effects could occur especially in fields like pain, whose treatment needs a comprehensive approach that involves physical, biochemical, and psychosocial factors (12–14).

In order to obtain a comprehensive analysis of the literature, the current review used the following search strategies in Pubmed:

- for VR and AR, the basic query was [“systematic review” (Publication Type)] AND (“virtual reality” OR “augmented reality”);
- for manual therapies, the basic query was [“systematic review” (Publication Type)] AND [bodywork OR (“manual therapy”) OR (“osteopathic manipulative treatment”) OR (“osteopathic treatment”) OR (“high velocity low amplitude”) OR (“muscle energy”) OR (counterstrain) OR (“myofascial release”) OR (craniosacral) OR (“cranial field”) OR (“lymphatic pump”) OR (“rib raising”) OR (“spinal manipulative”) OR (“suboccipital decompression”) OR (“fourth ventricle”) OR (CV4) OR (“trigger point”)].

For both queries, we repeated the search by adding condition-specific keywords, such as pain, chronic pain, balance, dementia, depression, quality of life, phobia, and surgery. We then selected the papers involving adult people (18+ years old), from inception to February 2021.

## The Current State of the Use of MTs, VR, and AR

MTs, VR, and AR are used to manage several pathological conditions and improve patients' health-related quality of life (HRQoL) and disability. Over the last few years, several systematic reviews investigated their efficacy and safety. Despite the need for more rigorous research—more significant samples, better description of randomization, allocation concealment, interventions, and control group, follow-up assessment, better statistical analyses, standardized methodology, uniform choice of the outcomes to easily pool the results in meta-analyses—for MTs (15–17), VR and AR (2, 10, 18), these interventions have shown positive effects on several conditions as reported in the next paragraphs. VR and AR also helped augment medical education and training (5, 19–22).

MTs, VR, and AR are generally safe—just some minor transient adverse effects were reported, particularly muscle stiffness and symptoms worsening for MTs (23, 24), and musculoskeletal pain, fatigue, and dizziness for VR and AR (25–28). However, due to the limitations mentioned above, authors had difficulties in drawing firm conclusions or recommendations for MTs (17, 29), VR, and AR (11, 18, 30, 31) as valid and reliable treatments.

### The Effects of MTs: A Summary

Evidence shows that MTs could help improve fatigue, sleep, and well-being (32–35). MTs seem to increase functionality and HRQoL, while reducing pain in pregnant and postpartum women with LBP, pelvic pain and dysmenorrhea (36–38). MTs might also positively affect maternal antenatal depression (39).

MTs could be useful in caring for individuals with acute and chronic pain. In particular, MTs (e.g., spinal manipulation and mobilization, massage, OMT) showed clinical effects in acute and chronic LBP, neck pain, lateral epicondylitis, headaches, pain due to surgical and non-surgical adhesion, and pain-related disability (17, 23, 24, 40–45). A recent meta-analysis found that OMT, in particular, myofascial release, is effective in reducing pain and improving functional status (even through the reduction of fear-avoidance beliefs) in case of non-specific LBP, even when assessed after 12 weeks (46). MTs seem to reduce pain even in other conditions such as temporomandibular disorder (47), irritable bowel syndrome (48), and fibromyalgia (49, 50).

Nevertheless, there are mixed results on the effectiveness of MTs in reducing pain: for instance, in some cases MTs such as spinal therapies seem to be ineffective in treating mild to moderate chronic LBP (51), whereas in other cases the effects remain clinically significant only in the short-term (e. g., weeks) (49, 50). Although the clinical significance of the result seems small, a recent randomized controlled trial found that, compared to sham therapy, OMT induced a higher reduction in LBP-specific activity limitations at 3 and 12 months (52).

Some weak evidence shows that MTs, for instance, OMT, might help prevent falls and ameliorate objective measures of mobility and balance (e.g., sit-to-stand, gait speed) in the case of dizziness (53) and cervical vertigo (54). MTs might also improve gait in the case of Parkinson's disease (PD) (55). However, in the elderly MTs seem to improve balance function only together with vestibular rehabilitation (56).

Lastly, some evidence shows MTs might help manage essential hypertension as an adjunctive therapy to drugs (15, 16), an effect that could be due to the potential ability of MTs to positively affect the autonomic nervous system regulation (57).

### The Effects of VR and AR: A Summary

Table 1 briefly summarizes the main findings that arise from the systematic reviews published during the years about VR and AR.

**Table 1:** A summary of the main findings of the systematic reviews about VR and AR.

**Field**	**References**	**VR/AR**	**Main findings**
Education	Uruthiralingam and Rea, (20)	VR/AR	Improved anatomical education for undergraduate and postgraduate students, residents, dentistry, and nursery students in 75 out of 87 reviewed papers.
	Zhao et al. (22) Kyaw et al. (19)	VR	Improved the test scores compared to other methods (e.g., lectures, textbooks, and dissections) in different anatomical fields (e.g., musculoskeletal, neurologic, and gastroenteric). Longer courses showed larger effect size than short ones. The more the learners could interact with the 3D virtual models, the more the gain in knowledge and in cognitive skills (e.g., history taking, counseling competencies, decision-making, and communication).
	Tang et al. (5)	AR	Improved test scores and higher satisfaction and learning engagement using MagicBook, an AR system that uses webcam or smartphone to recreate 3D interactive models.
	Quintero et al. (21)	AR	Increased the attention, interest and motivation of students, even with disabilities or special educational needs.
Oral and maxillofacial surgery	Joda et al. (4) Farronato et al. (58) Ayoub and Pulijala, (59)	VR/AR	Improved manual dexterity and surgical skills in undergraduate and postgraduate students. Improved the execution of several procedures, including caries and submandibular glands removal and orbital floor reconstruction. The simulations were able to detect students with potential learning challenges and to discriminate between novices and experts.
Surgery	Tang et al. (5) Barsom et al. (60) Wong et al. (61) Tang et al. (62) Meola et al. (63) Fida et al. (64)	VR/AR	Reduced intraoperative times, potential ischemic times, tissue damages in several medical procedures, including laparoscopic tasks, bone reconstruction, lumbar punctures, otorhinolaryngologic and neurosurgical operations. Facilitated and improved pancreatic, hepato-biliary, and urogenital surgery. Surgeons could manipulate the 3D virtual representation to assure to not expose or harm delicate tissues. The AR navigation system allowed to better view the anatomical structures, and reduced mental demand, physical demand, effort, and frustration compared to conventional navigation systems.
Psychology	Freeman et al. (2)	VRET, VR cognitive therapy	Improved specific phobias, social anxiety, PTSD, obsessive-compulsive disorder, generalized anxiety disorder, psychotic disorders (reduced distress and persecutory delusions, and improved social functioning), anorexia nervosa and cravings for substances. The effect sizes were comparable to face-to-face exposure therapy and quite large, and the results persisted for several years after the end of the therapy.
	Chicchi Giglioli et al. (65)	ARET	Improved phobia of small animals and acrophobia.
	Wechsler et al. (66)	VRET and VR cognitive therapy	Improved phobias (especially, specific phobia and agoraphobia) and anxiety more effective than inactive control groups (e.g., waitlist, placebo, or no treatment). Comparable to *in vivo* exposure or cognitive therapies. Slightly inferior for social phobia.
	Segawa et al. (67)	VRET	Mixed results for treating craving of several substances (i.e., nicotine, alcohol, cocaine, and cannabis) or behavior (i.e., gambling and internet gaming). Comparable to CBT in terms of effectiveness and relapse. A combination of VR, exposure, and cognitive therapy could be the best treatment course. Helped in eliciting cravings, thus allowing to comprehend which stimuli can trigger them.
	Fodor et al. (68)	VR and VRET	Reduced anxiety and depression more than control interventions (i.e., waitlist, placebo, relaxation), but similar to other psychological interventions.
	Eijlers et al. (69) Iannicelli et al. (70) Gujjar et al. (71) Chan et al. (72)	VR	Reduced anxiety and pain during medical procedures, including immunization, surgery, burn, dental, and oncological care, and venous access more than usual care (although the reviewed studies did not clearly describe usual care).
	Luo et al. (25)	VR	VR+analgesics for burn care (e.g., dressing change, and physical therapy) reduced unpleasantness, pain, the time spent thinking about pain, anxiety. VR was perceived as fun, even when the level of perceived presence was quite low (3.4 out of 10).
Physiotherapy and rehabilitation	de Amorim et al. (30)	VR	Improved static and dynamic balance, mobility, gait, and reduced sitting and standing time, fear and risk of falls in various elderly samples, healthy or with some disorder (e.g., balance deficit, diabetes mellitus, or PD) more than placebo, standard proprioceptive training, and kinesiotherapy
	Lee et al. (10) de Araújo et al. (18) Iruthayarajah et al. (26) Massetti et al. (28)	VR	Improved balance, stride length, sitting and standing time, when VR was used in rehabilitation programs for spinal cord injuries, limb and overall function in chronic stroke patients, PD, and multiple sclerosis. Improved aerobic and motor function, muscle tension, muscle strength, and activities of daily life alone or in combination with occupational therapy or physiotherapy. Some minor and transient adverse effects (e.g., musculoskeletal pain, fatigue, and dizziness) were reported.
	Ahern et al. (73)	VR	Reduced fear of movement in patients with LBP more than conventional stabilization exercises or physical therapy.
	Lei et al. (27)	VR	Improved HRQoL, level of confidence in difficult activities that could cause falls, and neuropsychiatric symptoms (i.e., anxiety and depression) more than standard care, conventional therapy, or any other non-VR rehabilitation program for PD.
	Manivannan et al. (74)	VR	Improved executive functions, driving attitude, attention, learning, and problem solving-skills in case of traumatic brain injury, but lack of improvement in employment rate.
	Pedroli et al. (75)	VR	Improved daily life in patients with USN. More useful than classical tests for assessing the severity of USN, since VR had the advantage of testing USN in simulated real-life conditions, e.g., driving in the streets.
Pain	Chi et al. (76)	VR	Reduced neuropathic pain in patients with spinal cord injuries through various VR systems (virtual walking, training, illusion, or hypnosis).
	Gumaa and Rehan Youssef, (77)	VR	Reduced chronic neck pain and shoulder impingement syndrome more than conventional therapy. VR was similar or inferior to exercises in many other conditions, including rheumatoid arthritis, knee arthritis, back pain, and fibromyalgia.
Pathophysiology	Bluett et al. (78)	VR	Improved understanding of the pathophysiology of freezing of gait in PD by reproducing this event in a safe environment (i.e., without the risk of a real fall).

*AR, augmented reality; ARET, augmented reality exposure therapy; CBT, cognitive behavioral therapy; HRQoL, health-related quality of life; LBP, low back pain; PD, Parkinson's disease; PTSD, post-traumatic stress disorder; USN, unilateral spatial neglect; VR, virtual reality; VRET, virtual reality exposure therapy*.

As an educational tool, VR and AR enable students to better understand anatomical structures and their spatial relationships through interactive 3D images or models (5, 19, 20, 22). AR also catches the attention of students with disabilities or special educational needs, enacting inclusive education (21).

Specific surgical-oriented VR/AR systems [such as the Da Vinci remote surgical system (79)] improve manual dexterity, surgical skills, and intraoperative time in several surgical fields (e.g., dental implantology, neurosurgical operations, and hepato-biliary surgery), in particular, together with interfaces for haptic feedback (4, 5, 58–64). Viewing the 3D reconstructions of the patient's tissues (previously assessed through imaging techniques) during the operations through AR, surgeons could be more accurate and avoid harming delicate tissues (5, 58, 60, 61, 63).

In the field of psychology, both VR and AR have been combined with exposure therapy—recreating the fear/anxiety-inducing stimuli (Virtual Reality Exposure Therapy, VRET, and Augmented Reality Exposure Therapy, ARET); and cognitive therapy—using a virtual coach voiced by a real therapist—to augment the treatment of different kinds of phobias and anxiety, cravings for various substances (e.g., cigarettes, cocaine), post-traumatic stress disorder (PTSD), depressive symptoms, and distorted body image in case of anorexia nervosa. The effects are often transferred successfully in everyday life and maintained for months or years, maybe because AR and (mostly) VR allow the simulation of an ecological environment where every exposition cue can be entirely controlled (2, 65–68). For social phobia, VR is slightly inferior to *in vivo* exposure therapy, possibly due to the difficulties in recreating credible social interactions (66) or the uncanny valley hypothesis—briefly, feeling eeriness and aversion toward characters/avatars that closely resemble humans but show “non-human” features (e.g., moving robotically or having “cold” eyes) (80, 81). Moreover, VR is useful to reduce anxiety and pain during medical procedures (e.g., immunization, surgery, and oncological care), thus acting as a powerful distraction (69–72)—the greater the immersive experience, the greater the effects (25).

Beyond the field of psychology, VR improves static and dynamic balance, mobility, gait, stride length, sitting and standing time, fear of movements and risk of falls, aerobic and motor function, muscle tension and strength, and activities of daily life in various populations, healthy or with some disorder (e.g., balance deficit, spinal cord injuries, stroke, PD, and multiple sclerosis) (10, 18, 26–28, 30, 73, 76). Moreover, VR reduces neuropathic pain in spinal cord injuries (27), anxiety, and depressive symptoms in people with PD (76). VR also improves executive functions in case of traumatic brain injury (74) and attention in people with unilateral spatial neglect (USN), a neurological disorder that commonly follows injuries (e.g., stroke) to one brain hemisphere and induces deficit in responding to stimuli placed on one side on the vision field (75). VR also helps reduce musculoskeletal related pain (e.g., chronic neck pain and shoulder impingement syndrome), although often similar or inferior to recommended exercises (77).

It is worth noting that, in both fields of psychology and rehabilitation, VR and AR are used mostly as add-on therapies combined with already established treatments. Indeed, VR and AR seem to enhance the conventional therapies' effects through the intensification of experience induced by gamification and realistic simulations (2, 10, 18, 30, 66).

Lastly, by recreating certain events (e.g., phobias and falls) in a simulated environment, VR and AR could also help understand the causes of psychological and neuromotor disorders and the stimuli that trigger them, allowing practitioners to deliver the therapy that best suits their patients (2, 75, 78).

## The Crossroads Between MTs, VR and AR

Despite the burgeoning research on VR and AR role in the rehabilitation field, MTs and their combination with these technologies seem to have not received attention. Research has focused primarily on patients experiencing VR and AR before or after physical therapy sessions, with therapists acting as “mere” support (e.g., supervision, safeguarding, and manual assistance) or whenever patients had difficulties in executing a task (82–85). Due to their reported effects, MTs could be combined with VR and AR to improve the management of several clinical conditions, including depression, chronic pain, neuropathic pain, and eating disorders.

**Figure 1:**
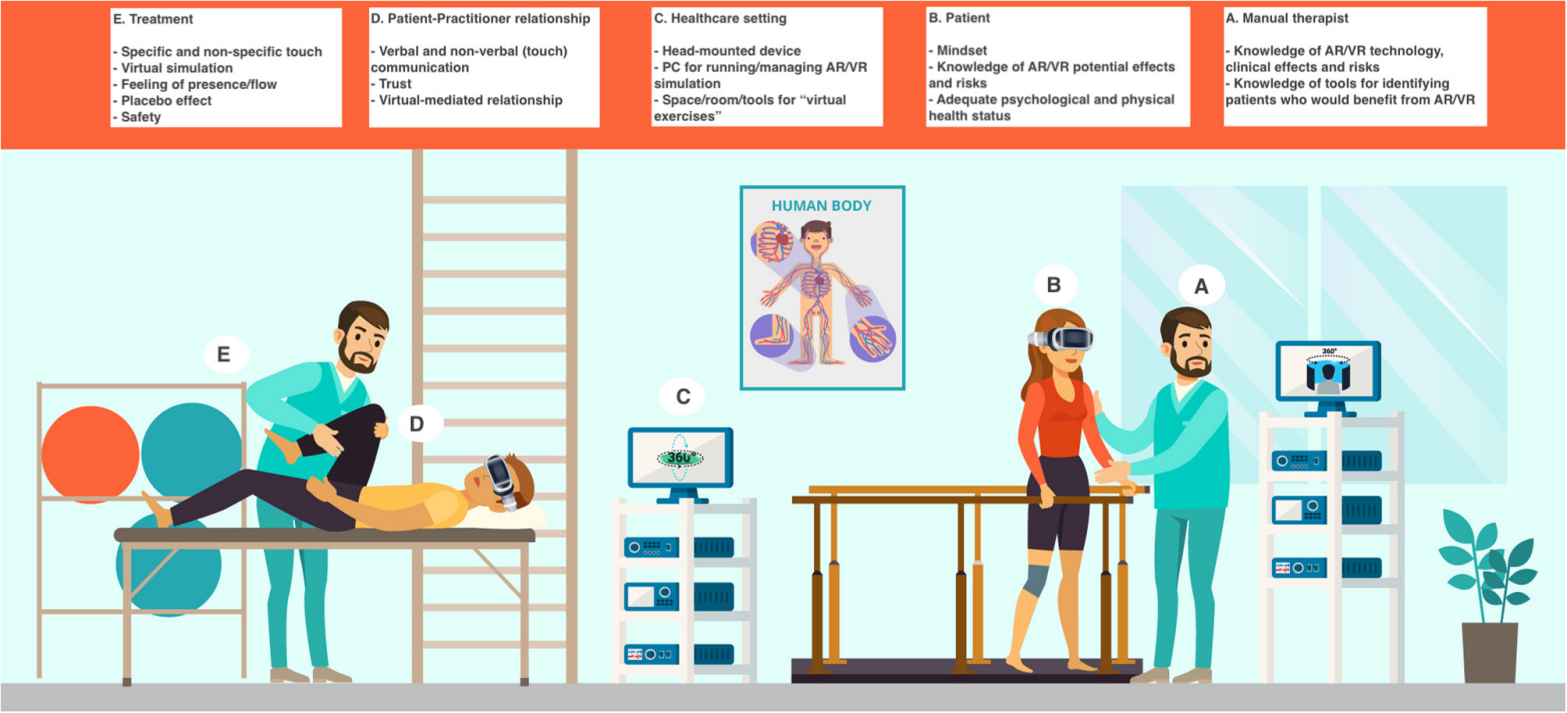
The integration of VR and AR in the MT setting. The MT setting would be enhanced by the addition of VR and AR: however, this implies the therapist and the patient need to pay attention to both old and new therapeutic factors. The factors could be summarized as follows (see the section “The Crossroads between MTs, VR and AR,” for further information). **(A)** Manual therapist's features: knowledge of AR and VR technology, clinical effects and risks; knowledge of tools (e.g., questionnaires, biomarkers such as heart rate variability) for identifying patients who would benefit from AR or VR (every patient could respond differently to them and some patients could be at a higher risk of experiencing adverse effects, from cybersickness to depersonalization). **(B)** Patient's features: mindset (i.e., expectation about VR or AR positive effects); knowledge of AR and VR potential effects and risks; adequate psychological and physical health status (based on their psychophysical health, some patients could be at a higher risk of experiencing adverse effects, from cybersickness to depersonalization). **(C)** Healthcare setting: head-mounted device; PC (or other types of hardware) for running/managing AR or VR simulation; space/room/tools for “virtual exercises.” **(D)** Patient-practitioner relationship: verbal and non-verbal (touch) communication; trust (it is paramount when the patient is immersed in the virtual environment and, therefore, does not see the “real” therapist); virtual-mediated relationship (in the virtual simulation, there could be an avatar of the therapist and phenomena like the uncanny valley could happen). **(E)** Treatment features: specific (technical) and non-specific (placebo) touch; virtual simulation; feeling of presence/flow (necessary for an optimal AR and VR experience); placebo effect (the patient could feel better just due to the simple fact that an “awesome” technology is being used); safety.

Considering that MTs elicit tactile, proprioceptive, and interoceptive sensations, whereas VR and AR send primarily visual and auditory stimuli, these interventions could be complementary. It is true that new technologies are emerging to bring tactile and internal sensations in simulated environments—for example, force feedback systems like Geomagic Touch to simulate tactile sensations and proprioception (86), skin-integrated wireless haptic interfaces to transfer touch (87), and wearable acoustic or vibrotactile transducers for evoking inner body sensations (88), but MTs could be the easiest way to add the sense of touch, proprioception, and interoception to VR and AR (89). On the other hand, VR and AR could be used as add-on therapies to enhance the effects of MTs in the same way they do when added to psychological and rehabilitative treatments (Figure 1).

The combination of MTs with VR and AR could define a whole-body intervention that includes both interoceptive and exteroceptive signals, with the potential of improving sensorimotor integration and rebalancing distorted perceptions and body images in patients suffering from eating disorders, autism spectrum disorder (ASD), or other disperceptive conditions (maybe even body identity integrity disorder) (88, 90, 91). Indeed, Suzuki et al. found that, through AR, the online integration of visual, tactile, and internal stimuli (i.e., the heart rhythm) enhances the sense of ownership in the case of rubber hand illusion (RHI) (92).

Therefore, after discussing the usefulness of low-cost head-mounted displays (HMDs), the devices more suitable to introduce VR in the setting of MTs, this section will focus on the potential and reciprocal contributions between VR (in particular), AR, and MTs to improve healthcare.

As a side note, VR and AR could also improve students' training and manual skills. For instance, AR-derived 3D models of the manipulated/mobilized body tissues could enhance the manipulations' efficacy and avoid potential adverse effects. However, the technology required for achieving the high precision in tactile and force reproduction needed to perform efficient training is still lacking at the moment (93).

### The Advantages of HMDs in Research and Clinical Fields

A limitation of several VR studies is that the authors used systems such as Nintendo® Wii Fit, PlayStation® 2 EyeToy camera, and Microsoft® Xbox Kinect that, although more engaging than conventional therapy, lack the immersion that should characterize VR—they lack presence (1, 2). Furthermore, they are non-specific systems for rehabilitation as they were designed for other purposes, and therefore, they may show lower therapeutic usefulness. Indeed, VR systems explicitly intended for clinical purposes showed more significant effects than non-specific ones and conventional therapy in the rehabilitation of post-stroke motor recovery (11). However, such systems can be costly and need advanced engineering to be realized (88).

The commercialization of low-cost HMDs, which can be associated with personal computers, tablets, and smartphones, partially overcome the lack of immersion (2). Indeed, HMDs could help elicit the feeling of presence through a stereoscopic view of the simulated environment, making the users focus on the virtual experience and avoid external (visual) distractions. HMDs continuously track the position of the user's head to allow a 360° visual navigation of the virtual environment: if turning the head failed to induce a change in the visual image, the users would immediately realize to be in a non-realistic environment and, thus, presence would be impossible (2). HMDs may also include headphones to send auditory stimuli to the users, thus monopolizing both eyes and ears (1, 2). However, visual and auditory stimuli are not enough to create a truly immersive experience, which also needs proprioceptive or haptic stimuli, especially when the simulation aims to target body ownership (1, 2, 88, 90). For this reason, HMDs might be combined with proprioceptive/haptic devices placed on various body parts (1) or real-time motion capture, although the cost for such a set-up could be high (88).

Despite the limitations regarding immersion, HMDs could represent the key for combining VR and AR with MTs: on the one hand, the tactile stimuli of MTs might somehow replace the proprioceptive and interoceptive stimuli needed for immersion; on the other hand, while receiving MTs, patients tend to stand still, e.g., lying on the massage table, and so tracking the body's movements could be unnecessary. Therefore, while receiving treatment, the patients could easily immerse themselves in the VR experience, hence benefitting from interventions. In fact, the therapeutic goal becomes paramount to choose the proper equipment to use: for instance, proprioceptive devices or motion-capture would be necessary if the VR experience had to directly act on body perception, whereas the same devices would be not required if the VR simulation served just as a distractor or relaxing stimulus.

HMDs could also enhance ecological validity in both the research and the clinical setting. In neuroscience, VR has been deemed paramount to introducing ecological validity in the laboratories while maintaining adequate control of experimental stimuli and confounding variables (94). Using VR, researchers may study a range of cognitive processes in a simulated environment that resembles everyday life—researchers may recreate realistic social interactions, emotional cues, and narrative experiences–, thus eliciting more natural behaviors and physiological reactions and allowing better findings generalization (94, 95). Creating an ecologically valid environment is also paramount in the rehabilitation field: patients could interact with controlled, personalized, and meaningful sensorimotor stimuli, while receiving real-time feedback about the performance through visual, auditory, or kinesthetic means (11, 96, 97).

The portability and accessibility of HMDs could increase the studies' ecological validity and generalization because researchers may study VR directly in the clinical setting or at the patients' homes, thus genuinely bringing VR outside the laboratories and into real life. Indeed, VR helps people perform therapeutic exercises at home with good results in stroke and incomplete spinal cord injury (98–102).

Relying on the Internet and the Cloud, HMDs and associated devices (e.g., smartphones) could send data about the performed exercises directly to the therapists, thus allowing them to supervise their patients better and potentially improving adherence to therapy (103). Even automated daily monitoring by home-based VR systems helps increase patients adherence (98); moreover, it is possible to create a multi-user virtual environment that allows patients and caregivers to interact among themselves to improve rehabilitation outcomes (101, 102).

All these features can also be used to customize the treatment received by the patients. Indeed, technological evolution allows therapists to model therapeutic exercises or tasks for their patients: therapists could record themselves while doing the exercises, save and customize the motions through VR software and, then, construct specific and easily flexible therapeutic plans. On the other side, patients could view the tasks to be performed as many times as they need, detect maladaptive movements (through kinesthetic or visual sensors) and check their performance and progress (97, 98, 103). The correction of maladaptive movements or postures could also be augmented by integrating kinesthetic sensors with output devices placed on various body parts. Whenever the sensors (e.g., accelerometers) detect a maladaptive behavior, the output devices could induce accurate vibrotactile stimuli on the body, prompting the users to adjust their posture or motion (104).

Manual therapists could instruct patients through HMDs as well, showing them how to perform several kinds of self-massage or self-touch that could help maintain the effects of MTs in the long-term. Regarding home-based care, the use of wireless haptic interfaces (87) could even allow therapists to perform MTs remotely (at least, those involving gentle or moderate strokings and pressures).

Although cheaper than other technologies, HMDs are not free: their cost could still be unaffordable for people of low socioeconomic status. Therefore, who should bear the cost of HMDs (or similar devices) for these people, should they need them for improving their health through home-based VR sessions (105)? Indeed, this is a central theme that needs to be faced by the healthcare system if VR and AR are to be introduced in clinical practice.

### How MTs Could Enhance VR and AR Experience

MTs may induce local effects, through the activation of mechanobiological pathways that can change the cells' behavior (106), and lead to systemic responses involving the circulatory, immune, endocrine, and nervous systems, and the mental state (6, 7, 9, 107–110). These systemic responses could influence the brain processes and, thus, interact with the VR and AR experience. In particular, MTs could facilitate the feeling of presence by influencing the physiological parameters (e.g., heart and breathing rate) that are typically regarded as markers of flow—the optimal experience of being fully immersed and involved in an activity perceived as immensely rewarding, with a good sense of agency and without self-referential thoughts (111). As flow entails a high degree of presence and concentration, eliciting such an experience could reduce cybersickness, which is negatively correlated to presence (although more rigorous studies are required since presence and cybersickness share common VR features, such as immersion) (3), thus improving VR efficacy.

Indeed, touch activates several neural pathways that project to cerebral areas involved in sensory, affective, and cognitive functions (112). Whereas, the Aβ nervous fibers send information about the sensory quality of touch principally, the Aδ and C fibers mediate affective touch, i.e., touch whose experience is accompanied by hedonic, emotional, and motivational qualities (89, 113). In particular, stimulating C fibers with slow (1–10 cm/s) and gentle (0.3–2.5 mN) strokings or pressures may induce a pleasant feeling and the activation of brain areas involved in interoceptive networks (e.g., insular and cingulate cortex) and emotional regulation (e.g., orbitofrontal cortex) (89, 114–116). The same neural activation occurs in other MTs like Swedish massage and spinal manipulative treatment (117, 118).

Interestingly, in the last few years, several papers showed that OMT may affect brain activity in both healthy adults (57, 119) and patients with chronic LBP (110, 120). OMT influenced regional cerebral blood flow, blood oxygen level dependent response and functional connectivity in many cerebrals areas, including the insula, cingulate cortex, amygdala, striatum, caudate, middle frontal and temporal gyri, cerebellum, and prefrontal cortex (57, 110, 119, 120). As all these areas play paramount roles in reorienting the attention between exogenous and endogenous stimuli, ideating and coordinating movement, monitoring the internal milieu, and regulating emotions, physiological arousal and pain (57, 110, 119, 120), affecting their activity might help people improve their experience in a simulated environment.

MTs may elicit a parasympathetic response, which usually correlates with a state of relaxation (6, 7, 57, 121, 122). MTs could reduce signal molecules tied to the stress response, including adrenocorticotropic hormone (ACTH), cortisol, and vasopressin, and enhance the production of endorphins, endocannabinoids, and oxytocin, hence favoring a more relaxed mind and a better mood (6, 7, 123–126). Touch might also increase trust in other people and prosocial behavior (127–129). These neuroendocrine and psychological effects could facilitate the immersion in the simulated environment, whether realized through VR or AR, and the flow experience. Although people need to activate stress and sympathetic responses to sustain the metabolic demands and the mental efforts required to accomplish specific VR or AR tasks (e.g., rehabilitation training), Tian et al. found indices of parasympathetic modulation (i.e., higher respiratory depth) in cases of flow (111). A stress response modulated by the parasympathetic tone could favor presence and reduce cybersickness, which is typically characterized by high secretion of hormones such as ACTH and vasopressin (3). Since parasympathetic is related to prosocial behavior, empathy, and a flexible mind (130, 131), MTs could improve the VR and AR efficacy in social phobia.

Currently, there is robust evidence that inflammation may influence the nervous system and cognitive-affective functions (132–135). Therefore, it is not surprising that an inflammatory challenge (typhoid vaccination) compromised spatial memory in a VR task, inducing an IL-6 increase that reduced glucose concentration within the perirhinal and entorhinal cortex and parahippocampal region (136). Since MTs might reduce inflammatory cytokines, including IL-1β, IL-2, IL-6, TNF-α, and INF-γ (123, 124, 137, 138), MTs could improve the performance obtained using simulations, thus augmenting the effect size of VR and AR interventions.

The activation of the interoceptive network and the physiological changes mentioned above, including the possible modifications of heart rate variability (HRV) elicited by the alteration in the autonomic tone (57, 116, 121, 122, 139), could be of great value to VR and AR since a solely exteroceptive illusion seems not enough to significantly reduce clinical pain (140). From recent studies, a more embodied illusion is needed: in particular, since we feel our body “from the inside,” VR and AR needs to create a simulation able to modulate the inner body sensations and feelings (e.g., heart rate) (88, 90). Therefore, MTs could help VR and AR create an interoceptive embodiment illusion without the need for complex devices, similarly to Suzuki et al., which showed an improved sense of body ownership with an AR-based integration of visual, tactile, and interoceptive stimuli (92). Moreover, a recent study showed that, after four OMT sessions, regional cerebral blood flow in areas such as the insula and the lentiform nucleus changed in correlation with HRV in patients with chronic LBP (110), thus showing how peripheral stimuli may affect brain activity. However, technological tools would be more precise than MTs in delivering specific stimuli. Besides, more research is required to assess whether MTs could effectively enhance the ownership of a virtual body (141).

### How VR and AR Could Improve MTs Effectiveness

VR and AR could help MTs in several ways and especially in treating pain and pain-related disability. Appropriate VR designs are recognized to provide analgesic outcomes (142): they can easily catch the attention of the users, shifting their cognitive resources away from their body to the virtual tasks, effects that can result in pain reduction (143). Considering that just thinking about a movement can trigger pain in some conditions (e.g., complex regional pain syndrome) (144, 145), VR and AR applications could recreate the stimuli that trigger pain in the same way they do with phobias and cravings (2).

A specific simulated environment depicting several types of postures, movements, or situations could help therapists and patients understand better when and how pain occurs. This type of simulation could show postures/movements from both first and third-person perspective: in the former case, appropriate kinesthetic and proprioceptive devices could be paramount to elicit the sense of body ownership efficiently; in the latter case, the observation of another person or avatar would trigger the activation of the mirror neuron system, an essential mediator for successful sensorimotor rehabilitation using VR (146–148).

That same simulated environment could also help decrease the pain-related experience in the same way that interventions such as RHI, mental imagery, and mirror therapy do (149–151). Indeed, VR and AR might revolutionize these and similar interventions by creating highly realistic immersive environments and reproducing a real embodied experience through the induction of visual, auditory, olfactory, tactile, and even interoceptive signals (88, 90).

Through VR and AR, the triggering stimuli can be adapted to patients' needs varying their intensity, duration, repetition, and so on. The simulated stimuli could even surpass reality (e.g., impossible body postures), hence favoring better results with more ease, although this is yet to be tested (2, 66). Indeed, VR and AR reduce pain in phantom limb pain (152) or complex regional pain syndrome (153).

All these effects could make patients more aware of their pain-related experience and improve pain management. For example, patients could better comprehend what events trigger their pain, thus learning how to manage and face them, and what events may reduce pain. On the other hand, therapists could better understand their patients' pain-related experiences. On occasion, pain treatment is problematic because patients fail to reproduce the exact conditions that induce their pain. Therefore, therapists lack a clear understanding of their patients' pain-related experience and the best intervention to perform. This situation is particularly significant when pain is not worsened by physical factors but by psychosocial ones, like stress, negative emotions, and beliefs. Despite evidence demonstrating that psychological and neurobiological factors can significantly influence musculoskeletal pain—pain is more about how the brain elaborates the psychophysical stimuli and responds to them, and that pain is, first and foremost, a subjective feeling (14, 154–156)—too often therapists give patients explanations in primarily biomechanical terms. Consequently, pain and disability persist, HRQoL decreases, and patients' costs to sustain for treatments rise (154, 155, 157–159).

VR and AR could help therapists enhance the effectiveness of pain neuroscience education and other programs aiming to teach the most recent discoveries about the complexity of pain (154, 160). Through virtual simulations, patients could see how the nervous system functions, understand the difference between simple nociception and complex phenomena like central sensitization that play a central role in chronic pain. Since alterations in the brain sensorimotor bodily maps are involved in chronic pain, VR could help patients actually see those maps. Moreover, therapists might show patients 3D interactive models of body anatomy, for instance, models of the intervertebral disks, thus removing false beliefs about anatomy (e.g., slipped disks) that could perpetrate pain through fear, nocebo effects, or other neural mechanisms (154, 155, 160, 161).

VR and AR applications could help patients overcome the fear of movement through exposition to activities perceived as painful (162, 163). While patients are distracted by VR applications, therapists could also reproduce supposed painful movements and then make patients aware that the movements were executed with little or no pain.

It is worth noting that sometimes pain could arise from traumatic events (e.g., car accidents) the patients fail to correctly remember—the memory was not adequately encoded and, therefore, the event lacks a precise context. A typical example of this situation is PTSD (164). The “blurred” memory could induce an overgeneralization of fear. Since the memory contains just general features of the traumatic event, fear is extended to every situation that shares those general conditions with the traumatic event (e.g., every time the subject gets in a car), increasing disability (164). A recent study found that, in this case, fear is not subject to extinction—the patients are not sure the exposed condition is the traumatic one and, thus, cannot change their behavior. A way to overcome that fear is by enriching the blurred memory, recreating in the therapeutic setting a “controlled” event similar to the traumatic one to skew fear toward this new context and, then, apply exposure therapy (164). VR could be perfect for this purpose and, since fear overgeneralization might sustain chronic pain (165), VR could reduce pain.

VR also has the potential to elicit awe, emotion at the core of experiences such as flow, strong spiritual and mystical feelings, and the “overview effect” (i.e., the sense of the interconnectedness of all life evoked in astronauts by the sight of Earth). Awe-inspiring environments might create a safe space that makes patients relaxed, peaceful, joyful, prosocially active, and ready to have a transformative experience, i.e., changing their behavior (166). This sense of safety and trust induced by VR could strengthen the placebo effect that arises from touch (touch is paramount for the social nature of humans) (89, 167), the therapeutic ritual intrinsic to MTs, and the relationship between therapists and patients, usually viewed as a cornerstone of MTs (168, 169). In the same way, VR could help people overcome the aversion they might have for touch, especially affective touch: indeed, patients who developed an insecure attachment style or experienced traumatic events, as well as patients who have PTSD, anorexia nervosa, ASD, or other conditions involving altered sensations and perceptions, rate affective touch as less pleasant than typical touch or even negative (170–173). Manual therapists might have difficulties in treating these people. A VR environment that induces peace and trust could conceivably elicit the same effects of oxytocin (usually correlated with trust), that is increasing tactile perception and, maybe, pleasantness (174, 175).

Lastly, as already described in the paragraph “3.1 The advantages of HMDs in research and clinical fields,” an advantage that VR and AR could give to MTs is the possibility of continuing and monitoring the treatment at the patient's home, thus improving adherence to therapy (103). By remotely connecting through the Internet, therapists could efficiently communicate with their patients, check whether they are performing the given exercises/tasks, and also evaluate how they are proceeding in the therapeutic plan. As therapists may record themselves to better show patients how to perform the therapeutic exercises, so the VR systems could record patients and send therapists their data—this would be particularly useful for those patients who can't reach the therapists' clinic, due to health-related disability or living far away from it.

However, this use of VR entails careful control and protection of patients' personal data to guarantee their privacy, especially if third parties are involved in the applications used by therapists and patients (176).

### The Brain, as A Bayesian Organ, Meets VR

The VR's putative modulatory effects on pain and other conditions (e.g., distorted body image) may be achieved since VR might reflect how the brain works (90). If the brain functions as a Bayesian organ that follows the free-energy principle, then the perception of both the inner and outer world arises from the brain—and arguably from the whole organism (14) —internal generative model, which is continuously used for efficiently adapting to the environment (177).

According to this view, the organism does not need to understand what is happening in the world perfectly, nor it could: due to the limited energy and resources (e.g., nutrients and cognitive capacity) available, it would be impossible to pay attention to all the environmental variables, i.e., sensory stimuli. Therefore, based on past sensorimotor activations (past beliefs), the brain creates a surrogate model of reality—the internal generative model—and then makes predictions about the upcoming events (posterior beliefs) by weighting that same model with the sensory evidence (actual information). These predictions must be accurate enough (not the most correct!) to allow the organism to survive and protect its psychophysical integrity. After every experience (and especially during sleep, when the brain is not engaged with the external world), the brain:

- tests its generative model against the sensory information coming from the world;
- tries to fit the sensory information in the generative model;
- if required, updates the generative model (we shall soon see how) to increase its usefulness and accuracy, but always trying to keep the model's complexity and redundancy at a minimum (177).

Since the resources available are limited, the brain needs to make useful predictions while maintaining a surrogate model of reality as simple as possible. This way, both the information free energy (the number of variables accounted for) and the thermodynamic free energy (the metabolic cost to encode and apply the generative model) are minimized [for an in-depth review, see (177)]. From a neural point of view, the brain tries to keep the number of synapses encoding the generative model at a minimum (indeed, during sleep, synaptic pruning occurs) to have simple and efficient neural networks able to respond to the world promptly and guarantee the organism survival. This process could be considered, briefly, the Bayesian idea of optimization applied to the brain (177).

The Bayesian brain has gained growing attention in the last years. It involves the whole body multisensorial, motor, and interoceptive/affective representation in the brain and its control by a complex neural network—the “body matrix” (88). Indeed, the generative model is an embodied simulation of the potential internal and sensorimotor states of the body. Based on the sensorimotor predictions made by integrating the generative model with the actual sensory information, the body matrix alters the physiological bodily conditions and the interoceptive and exteroceptive sensations through top-down modulation. Then, it redistributes throughout the whole organism the available energy and resources to cope with the upcoming events (88). But the brain has to minimize its free energy, i.e., the occurrence of “surprising” events not accounted for by the generative model. Indeed, the brain uses embodied simulations to represent and predict possible future sensations, actions, and emotions. These sensorimotor expectations may match or mismatch with the actual sensorimotor activity—in the latter scenario, a “surprise” or prediction error arises, which the brain must minimize to regulate the body and respond to it efficiently. As said, the brain could lack energy or not know how to manage the unexpected error (88, 90). Therefore, the brain may: (1) “suppress” prediction errors by superimposing its sensorimotor expectations on the body; (2) update its predictions, also changing the related beliefs about the world. However, it seems that the update of its internal generative model, i.e., learning, needs a great “surprise” and a “destabilized” sense of agency—the subject should fail to gather other information about the current situation or to enact the brain predictions, which means that usually, the brain superimposes “its” reality (88, 177, 178).

VR could facilitate the update of the internal generative model since it seems to function similarly to the brain: indeed, researchers aim to construct VR systems able to create sensory stimuli, detect the subjects' movements or actions and, based on a model of the users' body, try to predict the sensory consequences to give users a plausible and coherent experience, as if they were in the real world (90). If the brain is a Bayesian organ, then the process just described represents just what the brain does. On the other hand, the brain itself could represent a VR generating system that simulates a body and an environment to move around (177).

The more advanced the VR system is—i.e., multimodal devices to recreate every external and internal sensation, and software based on complex machine learning algorithms able to perform the aforementioned processes—the more the simulation is perceived as coherent and embodied. VR users, thus, would feel the virtual body as their own, the brain would generate its internal model based on it, and the sense of agency would become “embodied” in it (88, 90). Again, the simulation does not need to reflect reality, i.e., follow the physical laws of the real world or recreate the actual human body, to induce embodiment: as for presence, what matters is the coherence of the virtual simulation. Whenever the brain perceives coherence between the actions the user performs, the simulated events, and the sensations felt in response, the brain considers the situation as plausible and real, although potentially absurd (for instance, Steptoe et al. showed that it is possible to make people “own” and “control” a tail) (179).

When patients perceive a virtual simulation as coherent, therapists could use VR to elicit specific prediction errors through finely controlled stimuli to violate the internal generative model and facilitate its update. This way, therapists could favor modifying the dysfunctional representations of the body that may be at the root of pain, eating disorders, and other conditions that involve self-perception, including depression. Indeed, the brain uses the embodied simulation to predict sensations, actions, and even emotions (88, 90).

Therefore, complex VR simulations aim to act as cognitive prostheses to change the neurobiological processes that underlie both the perception and the physiological body responses to events. To this end, therapists need systems (hardware and software) that create a scenario that is felt coherent by the patients' brain, despite the awareness that “it is all an illusion,” and that is able to elicit specific responses (i.e., prediction errors) useful to reach the desired outcome (e.g., rehabilitation, pain reduction, better body image) (88, 90, 177). The more a VR system can do this, the better it is.

### The Limitations to the Integration of MTs With VR and AR

There are several considerations in regards to the integration between MTs and simulated experiences: in particular, they concern the state of research about the relationship between MTs and VR or AR, the characteristics of available VR and AR technologies, the possible negative consequences of their use.

Since literature lacks papers about the integration between MTs and VR or AR, trials that combine these interventions are required, starting from the ones that should assess the feasibility of combining the two approaches and evaluate possible adverse effects. The following trials should assess the effects of VR and AR applied before or after MTs sessions; others should investigate the effects of conducting VR sessions while the patient is receiving MT. Researchers should also evaluate whether VR could help patients change their counterproductive or wrong beliefs about pain and movement. Lastly, there is the need to understand whether manual therapists could use already developed VR and AR applications, or if new and original software is required.

Therapists need valid and reliable devices able to create immersive environments that induce presence/flow and avoid cybersickness. Since presence and cybersickness share a common ground—they are both increased by immersion, research should define which features of the simulation or the technology used could skew the virtual experience toward presence or cybersickness (3, 111, 180). In particular, some VR devices may favor cybersickness due to lack of accuracy in motion tracking or gesture recognition, low or not appropriate visual display frame rate, and even mismatches in conveying to the user different sensory information (especially, mismatches about visual-vestibular cues) (3).

Although useful as explained in “3.1 The advantage of HMDs in research and clinical fields,” HMDs might favor cybersickness, in particular, when the body is immobile while the eyes are tracking virtual visual stimuli. This might occur when the patient explores a virtual environment while receiving MT on a massage table (176, 181). In fact, manual stimulation could obstacolate the feeling of presence when it is not mirrored by something that is happening inside the virtual simulation: would it not be strange to feel touched but failing to see who or what is touching? If this were the case, the integration between MTs and VR or AR could be reduced to only specific and restricted interventions, which may require customized software that recreates the exact manual stimulations applied by the therapist. Should such a customized software be required, its cost could be particularly high and, therefore, therapists could have difficulties in acquiring it for their private practice.

According to some authors, HMDs could hinder the immersion in the virtual simulation due to the feeling of wearing HMDs (176). However, depending on the simulation, VR might act as a distractor powerful enough to make users forget about HMDs (as when people are lost in thought). On the other hand, wearing HMDs could help people remember they are experiencing a virtual simulation, that is discriminating between the real and virtual world, thus reducing possible adverse effects of VR (see below) (176).

Researchers could also study which biomarkers can discriminate between presence and cybersickness: for instance, some trials evaluated the use of HRV, which functions as a useful indicator of stress, sympathetic and parasympathetic modulation, all factors that can influence the balance between presence and cybersickness (3). The HRV measuring tools seem to be easily incorporated in the VR equipment and, from the preliminary results, several HRV metrics appear to detect cybersickness (182–184). The evaluation of cybersickness could also be tied to the research of biomarkers revealing the effectiveness of the virtual simulation in updating the brain generative model. Indeed, eliciting prediction errors (surprise) is necessary, but not sufficient for the update to take place: the whole simulation experience needs to be carefully constructed to properly activate the brain networks involved by the body matrix (88). Preliminary results showed some markers (e.g., increased pupil diameter and anterior cingulate cortex activity) might successfully detect the brain update of its beliefs: further proves would help create optimal virtual simulation (88, 185). Therefore, it becomes paramount to see whether MTs could enhance the feeling of presence, favor the update of the generative model and reduce cybersickness through their neuroendocrine effects (“3.2. How MTs could enhance VR experience”) or, as mentioned before, hinder presence, worsen the simulated experience and facilitate the negative effects VR and AR could have (see below).

Another possible issue with VR and AR is the uncanny valley phenomenon that could arise, for instance, when VR is used to support and supervise patients at home. The therapist avatar or, more likely, the virtual coach could elicit feelings of aversion if it should look quite-but-not-exactly human (80, 81), thus reducing both motivation and engagement in the VR therapy. The uncanny valley could induce a nocebo effect that would be deleterious in the long-run, especially for those conditions such as chronic pain, whose management needs to reduce any possible source of stress, anxiety, or fear (154, 159, 160). However, in the VR field, the uncanny valley seems to induce fewer avoidance feelings than expected (186), although more research is required to confirm this.

Another limitation is that VR simulations are not usually personalized for specific patients, but represent “generic” scenarios (e.g., events, avatars) to which every patient has to adapt. This limitation could entail a low engagement with the therapy, especially in the long-run and despite relying on specific features such as gamification for increasing motivation. Indeed, even factors such as age, gender, and personality traits may influence the VR experience and its effectiveness (97, 187). On the other hand, a therapy tailored to the patient's needs, preferences, and goals has long been recognized as paramount for the success and efficacy of any treatment plan, in particular, in case of chronic pain (159). Thus, VR software should allow therapists to create or use applications that could be easily customizable to offer calibrated and personalized interventions to their patients. The development of pathology-specific devices and software would help overcome these limitations and define precise treatment protocols, especially if both therapists and patients are actively involved in the development process itself (2, 11, 82). Despite this essential need, such software can be truly expensive to create (88), especially if we think about how every therapist and patient, as complex organisms, can behave differently based on the therapy used and have completely different experiences regarding the “same” symptom (e.g., pain) (14).

The addition of MTs to VR could increase the patients' motivation by improving their body awareness. Therapists can use touch to help patients become more aware of their bodily sensations—touch can elicit both interoceptive and proprioceptive feelings (89, 113) —and their meaning. Especially in case of pathological conditions, people do feel their body but do not know how to make sense of those “chaotic” sensations, thus becoming overwhelmed by them, e.g., people with lower interoceptive awareness show higher insular activation and greater neural processing (i.e., higher metabolic costs) than people with high interoceptive awareness (188). Therapists could then instruct patients to use their bodily feelings to develop better emotional awareness and regulation and guide them during the therapeutic process (189, 190). Consequently, patients could make better use of VR simulations, having been educated about managing the sensations that could emerge from their bodies. Besides, since touch elicits prosocial behavior and therapeutic compliance (127–129), it is conceivable that touch in itself could increase the patients' motivation and adherence to therapy. As interventions applied before a VR or AR session, MTs could overcome the before-mentioned limitations regarding the mismatch between real and simulated experience induced by an “unseen touch.” However, all these potential effects of MTs on VR experience are to be critically assessed.

Last but not least, a limitation regarding the use of VR and AR (especially VR) arises from the negative consequences the users could suffer. Patients could have difficulties in “returning to the real world” should the VR sessions be too frequent or take too much time (176). Motion sickness, nausea, dizziness, vomiting are some of the most common VR adverse effects, which can affect real life (e.g., driving a car) and last even for months for some patients (181). Besides, as VR could change for the better the body image of people, so VR could change it for the worse should the simulation not be carefully designed. By augmenting or intensifying the users' experience through specific stimuli, AR and VR could overload the users' neural and cognitive resources (the brain and the mind could receive too much information), thus increasing stress and inducing a strong “wear and tear” response (176, 191). By having their body swapped with a virtual one, patients could start having negative beliefs about their real body or even experiencing a distorted perception of it (105, 176). Indeed, Kilteni et al. found that even a short exposure to a virtual body illusion changes the corticospinal tracts' excitability, thus inducing cortical reorganization (192). The distorted perception could also affect the sensorimotor control of the real body and spatial navigation. For example, suppose the users experience a virtual body bigger than their own and complete hand-eye coordination tasks: in that case, they could have difficulties in hand-eye coordination once returned to their real body (191).

VR could also induce negative emotions that persist in real life—reliving a fear in a virtual environment could be way more intense than just thinking about it, and indeed immersion/absorption mediates the emotional experience in VR (193)—and cause memory alterations (e.g., did the event happen for real or just in VR?) (176). The same memory reconsolidation mechanism that VR might elicit for reducing fear, and associated pain (164), could negatively alter the patient's memory, especially when the simulation is immersive and realistic (176). Therefore, the patients could experience difficulties in discriminating between reality and virtual simulation, potentially suffering issues of depersonalization—the bodily self is perceived as unreal—or derealization—the external world is perceived as unreal. These problems could occur mainly in those people whose mental health is already fragile or at risk of deterioration (105), as it can be in the case of chronic pain (194). Besides, negative social interactions that the patients could have in VR, for instance, with the therapist's avatar, could lead to altered behavior in real life, thus with the real therapist (105, 176).

All these negative effects on emotion, memory, and behaviors may be increased by body-swapping since different bodies—different embodiments—seem to easily favor different emotions and behaviors that may transfer in real life (105, 176, 195). The virtual simulation could also induce negative effects on the sense of agency. If the virtual body's movements mismatched with the physical body's ones, the subjects could feel reduced control over their real body—this could be one of the aforementioned adverse effects of the integration between MTs and VR or AR. The consequences could be devastating, including depersonalization and feeling as an automaton (the body moves on its own) (105). Since creating a mismatch could be paramount to update the brain internal generative model, it becomes of the utmost importance to understand how to induce that mismatch without harming patients.

Therefore, beyond designing VR and AR technologies able to minimize all the risks mentioned above, it is paramount to help manual therapists understand which patients would benefit from VR and which patients would risk deteriorating their condition (105, 176, 191).

## Conclusions and Future Perspectives

The present review discussed the effects of MTs, VR, and AR. These interventions have been applied in several medicine and psychology fields and showed results that could significantly impact healthcare if confirmed by more rigorous trials. The commercialization of low-cost HMDs could allow manual therapists to combine VR and AR with MTs, thus creating an intervention that genuinely affects the whole mind-body unity. Indeed, MTs act primarily through touch, eliciting the tactile, proprioceptive, and interoceptive systems, whereas VR and AR send primarily visual-auditory stimuli and aim to affect the user's body's perception. Both MTs and VR may influence the mind, inspiring calm, joy, trust, and awe. Through remote Internet connection, HMDs would also allow manual therapists to supervise their patients at home and patients to continue their treatment outside the clinic, as it is already successfully happening in the rehabilitation field.

Regarding the effects the integration between MTs and VR or AR might have on balance and gait, both types of interventions have shown positive effects on these conditions, even in the case of pathologies such as PD (10, 55, 56, 82). Therefore, their integration could help improve stability, static and dynamic balance and reduce risk of falls by increasing body awareness through touch and augmenting cognitive functions related to motor skills through simulations. Moreover, as both inflammation and pain are linked to an increased risk of falls (196–198), especially in the elderly, MTs and VR or AR could also positively influence balance due the combination of the anti-inflammatory effects of MTs (6, 7, 123, 124) with the fear and pain modulation effects of VR and AR (2, 142, 143).

However, several limitations exist that must be overcome to fully harness the potential of the integration between MTs and virtual simulations, starting from assessing the feasibility of combining the two interventions. On the one hand, more research is required to see whether MTs could elicit or hinder the feeling of presence or flow during VR and augment the sense of ownership of the virtual body. On the other hand, more research is required to see whether VR and AR could help MTs manage painful conditions and address negative beliefs about movement and pain. There is also the need to evaluate which available VR and AR applications might be adequate to use in the MTs setting. Besides, to define a truly personalized approach, both therapists and patients have to be involved in elaborating VR and AR software and the process of gamification. Lastly, therapists require reliable tools to recognize which patients would benefit from VR and AR since, as any other treatment, they may induce serious adverse effects.

The success of the integration between MTs and VR or AR in everyday clinical practice will also depend on its practical feasibility. Indeed, although low-cost devices such as HDMs are ever more available (88), the software required for creating personalized applications could result in being particularly expensive for individual therapists without the support of healthcare institutions (4). Moreover, despite its use in rehabilitation and in the psychological field, literature lacks paper, whether controlled trials or systematic reviews, assessing the cost-effectiveness of the integration of VR and AR within the healthcare system (5, 22, 90). In the same way, literature lacks paper evaluating the adoption rate between patients: therefore, as of today, therapists cannot make a precise estimate about how many of their patients would use the potentially expensive and complex VR or AR systems they could buy for improving their practice (142).

All these questions are therefore left for future research, with the hope that healthcare and educational institutions may lead the innovation, both for their patients and the patients of private clinics, thus realizing the clinical usefulness of these interventions—with VR and AR we can make the impossible possible (2, 199).

## Author Contributions

FC, MA, and MC conceptualized the study. FC and MC drafted the initial manuscript. AG, VM, and AM critically reviewed the manuscript for important intellectual content. FC, MC, and JE revised the final manuscript. All authors approved the final manuscript as submitted and agree to be accountable for all aspects of the work.

## Conflict of Interest

VM was employed by company Softcare Studios. The remaining authors declare that the research was conducted in the absence of any commercial or financial relationships that could be construed as a potential conflict of interest.

## Abbreviations

ACTH: adrenocorticotropic hormone
ADHD: attention-deficit hyperactivity disorder
AR: augmented reality
ARET: augmented reality exposure therapy
ASD: autism spectrum disorder
HMD: head-mounted display
HRQoL: health-related quality of life
LBP: low back pain
MT: manual therapy
OMT: osteopathic manipulative treatment
PD: Parkinson's disease
PTSD: post-traumatic stress disorder
RHI: rubber hand illusion
VR: virtual reality
VRET: virtual reality exposure therapy.
